# 

**Published:** 1902-11-15

**Authors:** 


### ANNOUNCEMENTS.

### OHIO STATE DENTAL SOCIETY.

The thirty-sixth annual meeting of the Ohio State Dental Society, will be held in Columbus, December 2, 3, 4, 1902. This meeting promises to be one of the largest in the history of the society. Prominent members of the profession will present papers, and some of the most noted clinicians will operate. Arrangements have also been completed for one of the most extensive exhibitions of dental aids and appliances ever seen. All members of the profession are cordially invited to attend.

OTTO ARNOLD, Columbus, *President.*
S. D. RUGGLES, Portsmouth, *Secretary.*

### OHIO STATE BOARD OF DENTAL EXAMINERS.

The Ohio State Board of Dental Examiners will meet at the Neil House, Columbus, O., Nov. 25th, 26th and 27th, to examine applicants for registration. Applications should be filed by November 15th. For further particulars and application blanks address the Secretary, 112 East Broad Street, Columbus, O.

H. C. BROWN, *Secretary.*

### BOARD OF DENTAL EXAMINERS OF PENNSYLVANIA.

The Board of Dental Examiners of Pennsylvania will conduct examinations simultaneously in Philadelphia and Pittsburg, December 16–19, 1902. For examination papers or further information address Hon. James W. Latta, secretary Dental Council, Harrisburg, Pa. G. W. KLUMP, *Secretary.*

